# Disease awareness and healthcare utilization in rural South Africa: a comparative analysis of HIV and diabetes in the HAALSI cohort

**DOI:** 10.1186/s12889-023-17043-2

**Published:** 2023-11-08

**Authors:** Erika T. Beidelman, Till Bärnighausen, Coady Wing, Stephen Tollman, Meredith L. Phillips, Molly Rosenberg

**Affiliations:** 1 Department of Epidemiology and Biostatistics, Indiana University School of Public Health – Bloomington, 1025 E. 7th St, Bloomington, IN 47405 USA; 2 Heidelberg Institute of Global Health (HIGH), Heidelberg University, Heidelberg, Germany; 3 MRC/Wits Rural Public Health and Health Transitions Research Unit (Agincourt School of Public Health), Faculty of Health Sciences, University of the Witwatersrand, Johannesburg, South Africa; 4 Department of Global Health and Population, Harvard T.H. Chan School of Public Health, Boston, MA USA; 5 O’Neill School of Public and Environmental Affairs, Indiana University-Bloomington, Bloomington, USA; 6 INDEPTH Network, Accra, Ghana

**Keywords:** Healthcare expenditure, Healthcare utilization, HIV, Diabetes, Chronic Disease

## Abstract

**Background:**

Studies from rural South Africa indicate that people living with HIV (PLHIV) may have better health outcomes than those without, potentially due to the frequent healthcare visits necessitated by infection. Here, we examined the association between HIV status and healthcare utilization, using diabetes as an illustrative comparator of another high-burden, healthcare-intensive disease.

**Methods:**

Our exposure of interest was awareness of positive disease status for both HIV and diabetes. We identified 742 individuals who were HIV-positive and aware of their status and 305 who had diabetes and were aware of their status. HIV-positive status was further grouped by viral suppression. For each disease, we estimated the association with (1) other comorbid, chronic conditions, (2) health facility visits, (3) household-level healthcare expenditure, and (4) per-visit healthcare expenditure. We used log-binomial regression models to estimate prevalence ratios for co-morbid chronic conditions. Linear regression models were used for all other outcomes.

**Results:**

Virally suppressed PLHIV had decreased prevalence of chronic conditions, increased public clinic visits [β = 0.59, 95% CI: 0.5, 0.7], and reduced per-visit private clinic spending [β = -60, 95% CI: -83, -6] compared to those without HIV. No differences were observed in hospitalizations and per-visit spending at hospitals and public clinics between virally suppressed PLHIV and non-PLHIV. Conversely, diabetic individuals had increased prevalence of chronic conditions, increased visits across facility types, increased household-level expenditures (β = 88 R, 95% CI: 29, 154), per-visit hospital spending (β = 54 R, 95% CI: 7, 155), and per-visit public clinic spending (β = 31 R, 95% CI: 2, 74) compared to those without diabetes.

**Conclusions:**

Our results suggest that older adult PLHIV may visit public clinics more often than their HIV-negative counterparts but spend similarly on a per-visit basis. This provides preliminary evidence that the positive health outcomes observed among PLHIV in rural South Africa may be explained by different healthcare engagement patterns. Through our illustrative comparison between PLHIV and diabetics, we show that shifting disease burdens towards chronic and historically underfunded diseases, like diabetes, may be changing the landscape of health expenditure inequities.

**Supplementary Information:**

The online version contains supplementary material available at 10.1186/s12889-023-17043-2.

## Background

Widespread access to antiretroviral therapy (ART) has helped equalize health outcomes between people not living with Human Immunodeficiency Virus (HIV) and people living with HIV (PLHIV) globally [1–3]. In the setting of rural South Africa, evidence indicates that PLHIV may even present with better health outcomes than individuals not living with HIV [4–6]. Particularly, analyses of an older adult population in the rural Agincourt sub-district, northeast South Africa showed that PLHIV on ART had higher cognitive scores, lower systolic blood pressure, lower blood glucose levels, and greater use of hypertension treatment compared to people not living with HIV [4–6]. This evidence comes from the Health and Aging in Africa: A Longitudinal Study of an INDEPTH Community in South Africa (HAALSI) cohort which was designed to monitor health trends in an aging community. Within the rural and low-resource setting of the HAALSI cohort, these improved health outcomes could be related to the high level of healthcare system interaction necessitated by an HIV infection.

**Fig. 1:**
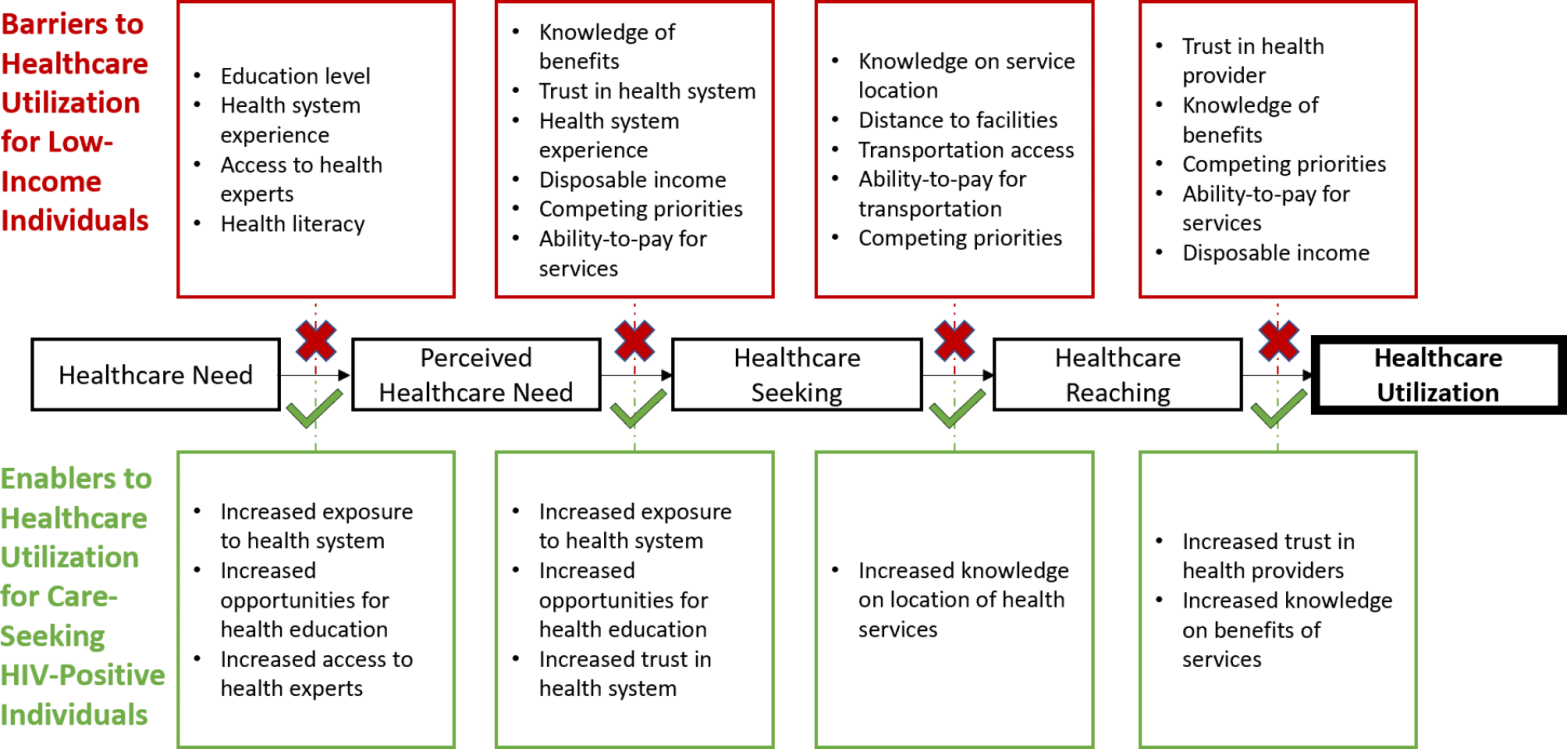
Conceptual model detailing barriers to healthcare utilization faced by low-income households and the enablers that can develop for care-seeking people living with HIV

Specifically, one hypothesis for these counter-intuitive findings is that due to the clinical needs of active HIV infections and the overall low access to health resources in the general population, PLHIV may develop greater knowledge of, and thus utilization of the formal healthcare system – even outside of HIV services. This greater utilization could, in turn, lead to better health outcomes. For example, evidence shows that attendance of at least one primary care visit a year is associated with increased likelihood of preventive measures with known health benefits like vaccinations, colonoscopies, and mammograms [7]. Other health gains documented from increased healthcare utilization are related to decreases in primary care sensitive conditions – conditions that benefit from preventive care, quick diagnosis, or regular management including chronic conditions like diabetes as well as infectious conditions like vaccine-preventable diseases [8–11]. Within the South African context, widespread expansion of ART has been associated with overall increases in public clinic utilization at a population-level [12]. Due to the double burden of disease and barriers to healthcare access in rural South Africa, it can be expected that increases in regular healthcare utilization may produce greater health benefits when compared to more developed countries [11]. Figure 1 summarizes the pathways through which HIV infection could increase primary and preventive healthcare utilization using Levesque’s healthcare access pathway [13–17]. Through this, evidence of improved health outcomes due to increased utilization in PLHIV could assist in arguments for primary and community care expansion in rural populations.

However, HIV is not the only highly prevalent chronic disease in South African older adults that requires frequent, long-term care. Type II diabetes (T2D) is another such example. Prevalence of T2D has been increasing over time for higher age groups in South Africa, reaching a prevalence of 12.4% among those 70 years and older, and is expected to increase further as the aging population expands [18, 19]. In the HAALSI cohort, there is an overall prevalence of diabetes between 7% and 12% depending upon the definition used [20]. However, as diabetes is only recently emerging as a priority area for healthcare funding in low-resource contexts, there is still lower access to care and subsidies for treatment compared to HIV [21–23]. Individuals with diabetes have also been reported as having lower quality of life and greater disability compared to their diabetes-free counterparts [18]. This contrasts with the preliminary evidence described above for older individuals with HIV.

To provide insight on the patterns of positive health outcomes observed across PLHIV in rural South Africa, this analysis used data from the HAALSI cohort to investigate the association between HIV+ status awareness, disease outcomes, and healthcare expenditures through separate but related analyses. Additionally, we conducted the same analyses for individuals with and without diabetes as an illustrative comparator of a different high-burden, chronic disease with lower levels of cost subsidization. We employed a cross-sectional design to assess these relationships in a group of rural adults aged 40 and older with high HIV and diabetes prevalence. We used measures of healthcare expenditure and self-reported healthcare visits to determine if there was evidence of differential patterns between care-seeking individuals with disease versus those without. Our objective was to compare health and healthcare utilization outcomes between individuals living with two highly prevalent chronic conditions (HIV and diabetes).

## Methods

### Study setting and population

The population for this cross-sectional secondary analysis is a part of the ongoing, longitudinal HAALSI cohort. Cohort participants were 40 years or older at the time of study enrollment in 2014 and resided in the rural Agincourt research site in the Mpumalanga province in northeast South Africa [20]. The Agincourt sub-district consists of 31 villages and an estimated 116,000 inhabitants [24]. This area is characterized as a previously Bantustan community under apartheid and, despite recent improvements, still has high rates of unemployment, high reliance on remittances from labor migrants and government support for income, high fertility rates, and lower access to key infrastructure compared to the general population [20, 24]. Within the HAALSI cohort there was a high rate of HIV, low rate of employment, and low rate of formal education [20]. The high rate of HIV within this community presents a unique opportunity to examine differences in PLHIV and people not living with HIV at the population-level.

The HAALSI cohort was designed to monitor health trends in an aging sub-population within the context of South Africa’s ongoing demographic and epidemiologic transitions [20]. Households were randomly sampled for participation using the Agincourt Health and Sociodemographic Surveillance System (HDSS) with an 86% response rate for Wave 1 [25]. Following enrollment and consent, household respondents participated in Wave 1 in-person interview surveys and voluntary biomarker testing [26]. HIV and ART laboratory testing was performed via dried blood spots for consenting participants [25]. Data used in this analysis were collected during Wave 1 of HAALSI from November 2014 to November 2015. The complete HAALSI cohort consisted of 5059 men and women aged 40 years and above at baseline [20]. Our sample (n = 4358) was restricted to individuals who had either self-reported or laboratory-confirmed HIV status information as well as self-reported data on annual household expenditures and healthcare utilization. Participants missing any of these data points were excluded from analysis as, without this data, exposure and outcome measures could not be constructed.

Healthcare in South Africa is delivered through both the public (government-sponsored) and private sectors. Approximately 80% of the population accesses services in the public sector where primary care services are delivered free of charge to any South African citizen [22, 27, 28]. Private clinics are generally considered to have more reliable stock of treatments and shorter wait times compared to public clinics but are generally more expensive [27]. Therefore, private clinics are frequented more often by individuals with higher socioeconomic status.

PLHIV are provided with free care and treatment in South Africa and progress through the HIV care cascade which is closely linked to general healthcare utilization. South Africa has a robust and well-developed ART program where individuals move through a potentially cyclical care cascade including testing, linkage to care, retainment in care, ART initiation, adherence to ART, and viral suppression [29, 30]. Though some efforts have been made to integrate HIV and other chronic disease care like diabetes, the care cascade for diabetes remains under-developed compared to that of HIV [31, 32]. The scale-up and availability of diabetes care in public clinics has been slow and not fully integrated in rural areas [21, 22]. While diabetes treatment is provided for free, it only includes medications listed on the Essential Medicines List which may frequently be out-of-stock in rural areas [21, 22, 27]. If resources for diabetes care are not available at the clinic level, patients are commonly referred to tertiary facilities which can increase patient costs and the likelihood for loss to follow-up [21]. In contrast, reports have shown more stable availability of HIV care and treatment at the primary care level as well as the integration of several policies to increase treatment adherence [21, 33].

### Key variables: exposures

The main exposure of interest in this study was being HIV + aware, defined as PLHIV displaying evidence that they are aware of that status (n = 742). Within interviews, participants were asked to provide a self-report of their HIV status and to consent to participate in laboratory HIV testing. Both were voluntary. Individuals were coded as ‘1’ if they either (a) self-reported as HIV + OR (b) self-reported as negative or declined to self-report but had positive laboratory tests for both HIV and ART. We chose to include people fulfilling the second set of criteria even if they did not self-report as HIV + because the presence of ART indicates that they are care-seeking. The HIV and ART laboratory measures provide more objective results to supplement the self-reported question that is subject to stigma and social desirability bias. Individuals were coded as ‘0’ for HIV + aware if they had (a) a negative laboratory test OR (b) a positive laboratory HIV test, a negative laboratory ART test, and a negative HIV self-report. We refer to this group as HIV-/status unaware. Participants who both did not self-report and did not consent to laboratory testing were excluded from the analysis. We chose to categorize individuals with evidence of HIV + status awareness because our pathways of interest are based in care-seeking behaviors that only occur once the positive HIV status is known. Hypothesized pathways were not based in any biological mechanism that comes from HIV seroconversion. Additionally, the HIV + aware group was further sub-divided into individuals whose laboratory testing indicated that they were (1) virally suppressed or (2) not virally suppressed. For the comparison analysis with diabetes as the exposure, we defined diabetes as a self-report of diagnosis by a healthcare provider (yes = 1, no = 0) (n = 305). We used these criteria instead of laboratory blood glucose testing to align with our HIV + aware definition and limit the exposure to individuals with evidence of care-seeking behaviors. Due to data limitations, this includes both T2D and Type 1 diabetes diagnoses and, thus, will be referred to as diabetes.

### Key variables: outcomes

We examined multiple healthcare outcomes to create a rich picture of healthcare utilization and spending in our population. These outcomes included:

1. Self-reported healthcare expenditure at the household-level (continuous, total Rand): During HAALSI surveys, the household respondent was asked to estimate the total amount in Rand that the household spent in the last year for 18 categories. We summed the self-reported household spending from the categories related to formal healthcare services including doctor’s, nurse’s, dentist’s, clinic, and hospital fees as well as medications, supplies, and other pharmacy purchases to estimate annual healthcare expenditure at the household-level. This healthcare expenditure variable does not include spending on transportation to the clinic or spending on traditional medicine services. Because HIV-related care and treatment is available at no charge within public facilities in South Africa, this analysis considers healthcare expenditure to encompass services outside of HIV [34].
2. Self-reported healthcare expenditure at the respondent & per-visit level (continuous, total Rand): Respondents who reported visiting a public clinic, private clinic, or hospital were asked to enumerate their total spending for their last visit at each relevant facility type. Total spending was then summed across each facility type to estimate per-visit, respondent-level spending that included accommodation, facility fees, food, medicines, phone expenses, and transportation. Individuals who reported no healthcare visits were excluded from the analysis to limit the analysis to care-seeking individuals.
3. Self-reported healthcare utilization (continuous, number of visits per facility type): Household respondents were also asked to self-report the number of times they visited public and private health clinics in the last three months as well as hospital admissions in the last 12 months. Public clinics refer to government-run facilities where many services are provided free-of-charge while private clinics refer to for-profit facilities run outside of the government health system. These measures were used to estimate healthcare utilization at the respondent-level as the total number of reported visits across public, private, and hospital facilities (separately).
4. Prevalence of suspected comorbid chronic conditions (binary indicators): Binary indicators of prevalence were created for hypertension, dyslipidemia, cognitive impairment, and obesity using results from cardiometabolic, laboratory, and cognitive testing. These conditions were chosen based on data availability in HAALSI as well as their presence as common emerging conditions within the aging population.

### Key variables: covariates

We also used data on several covariates to adjust for confounding and contextualize our findings. The covariates were age (in years), sex (male or female), household-level socioeconomic status (SES) (measured by asset-based wealth index quintiles), household size (number of residents), number of children in the household, highest education level (none, some primary, some secondary, or secondary or more), current employment (yes or no), and total annual household spending (in Rand). The wealth index quintiles were derived from a continuous asset index created with Agincourt HDSS and HAALSI questions related to household characteristics and asset ownership. A principal components analysis was then used to estimate the continuous asset index [20].

### Statistical analyses

Descriptive analyses were performed to determine differences in demographic and household characteristics between the HIV + aware and HIV-/status unaware groups as HIV status was our primary exposure of interest. These characteristics included respondent age, household size, number of children, respondent sex, respondent education level, respondent employment status, and household wealth index. For binary and categorical variables, a chi-squared test was performed to assess between-group differences. Continuous variables were assessed for between-group differences using the standard t-test except for the continuous household-level spending variable which was assessed with Mann-Whitney U-tests due to the left-skewedness.

We used log-binomial regression models to estimate separate prevalence ratios for the four identified co-morbid conditions (hypertension, dyslipidemia, cognitive impairment, and obesity) in individuals who were HIV + aware and virally suppressed and HIV + aware and virally unsuppressed compared to HIV-/status unaware. The same analysis was performed to compare co-morbid condition prevalence across individuals with and without self-reported, provider-diagnosed diabetes. The HIV-/status unaware and no diabetes groups, respectively, served as the reference group in these analyses. In instances where the log-binomial models did not converge, modified Poisson regressions with robust standard errors were used [35]. Next, we fit linear models to regress the average number of self-reported clinic visits (across each public clinics, private clinics, and hospitals) on HIV + status awareness and diabetes diagnosis separately. Finally, we fit linear models to regress each healthcare spending outcome (household-level total spending and respondent-level per-visit spending) on both HIV + status awareness and diabetes status separately. All specified models were adjusted for the respondent’s sex, respondent’s age, respondent’s education level, and household-level wealth index. Any conversions from Rand to USD are presented as an estimate of the approximate conversion at the time of May 2015, the midpoint of the data collection period. Conversions were made using a currency conversion application and facilitate understanding around the magnitude of spending. All statistical analyses were conducted using R version 4.1.1.

## Results

### Study population

Respondents in our sample (n = 4358) reported a median annual household expenditure of R 9,480 [IQR: R1605 to R8030] (~ 799 USD) while the median annual healthcare expenditure accounted for approximately 8% [IQR: 0–10%] of annual spending. See Figure S1 for a depiction of household annual and healthcare expenditures across wealth index quintiles. Over 22% of the total sample were laboratory-confirmed as HIV+, with 17% of those with laboratory-confirmed HIV meeting our definition of being aware of their positive status. 7% of the sample self-reported a provider diagnosis of diabetes. The HIV + aware and HIV-/status unaware groups were found to differ across the distribution of all characteristics except sex (see Table 1). Respondents classified as HIV + aware tended to live in households of a smaller household size and with fewer children. They were also younger, had higher levels of education, and were more likely to be of a lower wealth index.

**Table 1:** Comparison of household and sociodemographic characteristics of the HAALSI sub-population across the HIV- /status unaware and HIV + status aware groups

	Total (N = 4358)	HIV-(N = 3616)	HIV+ (N = 742)	
	Mean (SD)	Mean (SD)	Mean (SD)	P-Value
Age	61.4 (12.9)	62.7 (13.1)	55.3 (9.91)	< 0.001***
Household Size	5.23 (3.27)	5.32 (3.31)	4.81 (3.03)	< 0.001***
Number of Children	4.84 (2.62)	4.91 (2.64)	4.51 (2.48)	< 0.001***
Household Spending (Rand)^a^	9480 (23,000)	9770 (23,100)	8090 (22,400)	0.02*
	Freq. (%)	Freq. (%)	Freq. (%)	P-Value
Sex				0.8
*Male*	1918 (44.0%)	1587 (43.9%)	331 (44.6%)	
*Female*	2440 (56.0%)	2029 (56.1%)	411 (55.4%)	
Highest Education Level				< 0.001***
*None*	1924 (44.1%)	1636 (45.2%)	288 (38.8%)	
*Some Primary*	1512 (34.7%)	1250 (34.6%)	262 (35.3%)	
*Some Secondary*	519 (11.9%)	399 (11.0%)	120 (16.2%)	
*Secondary or more*	392 (9.0%)	322 (8.9%)	70 (9.4%)	
Employed				< 0.001***
*Yes*	703 (16.1%)	546 (15.1%)	157 (21.2%)	
*No*	3655 (83.9%)	3070 (84.9%)	585 (78.8%)	
Wealth Index				0.02*
*Quintile 1*	888 (20.4%)	713 (19.7%)	175 (23.6%)	
*Quintile 2*	880 (20.2%)	722 (20.0%)	158 (21.3%)	
*Quintile 3*	849 (19.5%)	704 (19.5%)	145 (19.5%)	
*Quintile 4*	848 (19.5%)	708 (19.6%)	140 (18.9%)	
*Quintile 5*	893 (20.5%)	769 (21.3%)	124 (16.7%)	

^b^Total annual household spending includes long distance travel, wedding expenses, birthdays, funerals, festivals, education, insurance premiums, home maintenance, vehicle service charges, taxes/fees/registration to government, healthcare, loan repayments, donations, and other expenses

### Prevalence of suspected co-morbid conditions

Being HIV + aware and virally suppressed was significantly associated with a 21% lower prevalence of hypertension [PR = 0.79, 95% CI: 0.71, 0.87], 24% lower prevalence of dyslipidemia [PR = 0.76, 95% CI: 0.66, 0.86], 36% lower prevalence of cognitive impairment [PR = 0.64, 95% CI: 0.40, 1.01], and 31% lower prevalence of obesity [PR = 0.69, 95% CI: 0.58, 0.82] compared to being HIV-/status unaware (see Table 2). No significant differences in the prevalence of dyslipidemia or cognitive impairment were observed when comparing the HIV + aware and virally unsuppressed group to the HIV-/status unaware group. However, the unsuppressed group was significantly associated with a 21% lower prevalence of hypertension [PR = 0.79, 95% CI: 0.68, 0.91] and a 36% lower prevalence of obesity [PR = 0.64, 95% CI: 0.49, 0.84] compared to the HIV-/status unaware group. In contrast, diabetes diagnosis was significantly associated with a 29% higher prevalence of hypertension [PR = 1.29, 95% CI: 1.23, 1.37], a 37% higher prevalence of dyslipidemia [PR = 1.37, 95% CI: 1.23, 1.53], and a 31% higher prevalence of obesity [PR = 1.31, 95% CI: 1.15, 1.49]. No significant difference in the prevalence of cognitive impairment was observed between diabetics and non-diabetics.

**Table 2:** Adjusted prevalence ratios of co-morbid conditions across HIV status awareness groups and diabetes status groups compared to disease-free individuals

	Hypertension PR [95% CI]	Dyslipidemia PR [95% CI]	Cognitive Impairment PR [95% CI]	Obesity PR [95% CI]
HIV-/Unaware	Ref	Ref	Ref	Ref
HIV + Aware, Sup.	**0.79 [0.71, 0.87]**	**0.76 [0.66, 0.86]**	**0.64 [0.40, 1.01]**	**0.69 [0.58, 0.82]**
HIV + Aware, Unsup.	**0.79 [0.68, 0.91]**	0.88 [0.74, 1.05]	0.90 [0.47, 1.72]	**0.64 [0.49, 0.84]**
Not Diabetic	Ref	Ref	Ref	Ref
Diabetic	**1.29 [1.23, 1.37]**	**1.37 [1.23, 1.53]**	1.12 [0.78, 1.60]	**1.31 [1.15, 1.49]**

Adjusted for respondent’s age (years), respondent’s sex (male/female), respondent’s education level (none, some primary, some secondary, or secondary or more), and household wealth quintile

### Healthcare utilization

Virally suppressed and unsuppressed HIV + aware individuals were associated with 0.59 [β = 0.59 visits, 95% CI: 05, 0.7] and 0.74 [β = 0.74 visits, 95% CI: 0.6, 0.9] more public clinic visits respectively in comparison to HIV-/unaware individuals (see Table 3). There were no differences in average private clinic visits in the last three months across the three HIV groups. HIV + aware and virally unsuppressed individuals were associated with 0.14 [β = 0.14 visits, 95% CI: 0.0, 0.3] more hospital admittances compared to HIV-/unaware individuals while HIV + aware and virally suppressed individuals were associated with no difference. Individuals diagnosed with diabetes were associated with 0.23 [β = 0.23 visits, 95% CI: 0.1, 0.3] more hospital admittances, 0.72 [β = 0.72 visits, 95% CI: 0.6, 0.9] more public clinic visits, and 0.14 [β = 0.14 visits, 95% CI: 0.0, 0.3] more private clinic visits compared to individuals with no diabetes.

**Table 3:** Estimated difference in average number of facility visits in the last three or 12 months across HIV status awareness groups and diabetes status groups

	Hospital, 12 m β [95% CI]	Public Clinic, 3 m β [95% CI]	Private Clinic, 3 m β [95% CI]
HIV-/Unaware	Ref	Ref	Ref
HIV + Aware, Sup.	-0.02 [-0.1, 0.1]	**0.59 [0.5, 0.7]**	0.01 [-0.1, 0.1]
HIV + Aware, Unsup.	**0.14 [0.0, 0.3]**	**0.74 [0.6, 0.9]**	-0.01 [-0.2, 0.2]
Not Diabetic	Ref	Ref	Ref
Diabetic	**0.23 [0.1, 0.3]**	**0.72 [0.6, 0.9]**	**0.14 [0.0, 0.3]**

Adjusted for respondent’s age (years), respondent’s sex (male/female), respondent’s education level (none, some primary, some secondary, or secondary or more), and household wealth quintile

### Healthcare Expenditure

For the HIV + status awareness analysis, modestly higher household-level healthcare expenditures were observed in HIV + status aware and virally suppressed groups compared to the HIV-/status unaware group (see Table 4). However, confidence intervals were relatively wide and approached the null [β = 30, 95% CI: 2, 66]. No difference was observed between HIV + aware and virally unsuppressed individuals compared to HIV-unaware individuals. Additionally, no differences were observed in the average reported per-visit cost for hospital visits and public clinic visits across the three HIV awareness groups. Being virally suppressed and unsuppressed HIV + aware was associated, respectively, with 60 Rand less [β = -60 Rand, 95% CI: -83, -6] and 73 Rand less [β = -73 Rand, 95% CI: -93, -2] spent on their last private clinic visit in comparison to HIV-/unaware individuals. Having a diabetes diagnosis was associated with significantly greater household-level annual healthcare spending [β = 88 Rand, 95% CI: 29, 154], per-visit hospital spending [β = 54 Rand, 95% CI: 7, 155], and per-visit public clinic spending [β = 31 Rand, 95% CI: 2, 74] compared to those without a diabetes diagnosis. No significant difference was observed for per-visit spending on private clinic visits.

**Table 4:** Effect estimates of HIV + status awareness groups and diabetes status groups on annual household healthcare expenditure and participant per-visit expenditure (in Rand)

	Annually^a,^^c^ β [95% CI]	Last Hospital Visit^b,^^c^ β [95% CI]	Last Public Clinic Visit^b,^^c^ β [95% CI]	Last Private Clinic Visit^b,^^c^ β [95% CI]
HIV-/Unaware	Ref	Ref	Ref	Ref
HIV + Aware, Sup.	**30.0 [1.8, 66.1]**	-17.6 [-54.2, -48.1]	21.9 [-4.2, 55.2]	**-60.1 [-83.0, -6.4]**
HIV + Aware, Unsup.	11.4 [-22.3, 59.7]	28.2 [-40.5, 176.3]	31.0 [-7.0, 84.5]	**-72.6 [-92.7, 2.3]**
Not Diabetic	Ref	Ref	Ref	Ref
Diabetic	**88.0 [39.2, 153.9]**	**53.6 [-7.4, 154.8]**	**30.9 [-1.7, 74.2]**	-9.1 [-57.5, 94.5]

^a^Annual healthcare spending includes doctor’s fees, nurse’s fees, dentist fees, clinic fees, hospital fees, medications, bandages, supplies, and other pharmacy/chemist purchases ^b^Per visit spending includes accommodation, facility fees, food, medicines, phone expenses, and transportation ^c^Estimates adjusted for log of annual household spending, respondent’s age (years), respondent’s sex (male/female), respondent’s education level (none, some primary, some secondary, or secondary or more), and household wealth quintile

## Discussion

Overall, we found that being HIV + aware and virally suppressed was associated with decreased prevalence of the selected co-morbid chronic conditions, increased public clinic visits, marginally increased household-level healthcare spending, but reduced per-visit private clinic spending compared to HIV-/status unaware individuals. Importantly, this pattern differed in key areas for individuals who were HIV + aware, but virally unsuppressed. Being status aware but virally unsuppressed was not associated with increased household-level healthcare spending but was associated with a greater number of average hospital visits compared to HIV-/unaware individuals. In stark contrast, being diabetic was associated with increased prevalence of selected co-morbid conditions, increased visits across all facility types, and increased spending at the household-level and per-visit level for hospital and public clinic visits compared to those without diabetes.

Our findings provide important context to prior reports from the HAALSI cohort of better health outcomes in PLHIV compared to participants without HIV [4, 5]. Virally suppressed PLHIV had higher household-level healthcare expenditure and greater public clinic visits, but similar costs per-visit compared to participants without HIV. This suggests that virally suppressed PLHIV may be receiving more healthcare overall and deriving from it measurable health benefits. Several plausible pathways exist for this relationship including early detection of co-morbid conditions, regular management and monitoring of co-morbid conditions, increased health literacy, and improved adherence to treatment regimens. The healthcare they are spending on is unlikely to be solely HIV care and treatment as this is offered for free in South Africa [34]. Additionally, we observed more public clinic visits but similar hospitalization numbers, indicating the increased care may be associated with primary and preventive rather than emergency or illness-based care. However, due to the limited detail in the healthcare utilization data, we cannot determine if the public clinic visits were used for services beyond standard HIV treatment.

Overall, the annual household and healthcare expenditure estimates we report were lower than those observed in previous research from Mutyambizi et al., (2017) which explored expenditures among South African diabetic patients [36]. We believe this is likely due to differences in source populations and sample selection methods. For instance, the HAALSI population has a higher unemployment rate and is a population-representative sample compared to the facility-based sample used in Mutyambizi et al. (2017) [36]. Additionally, previous research has found PLHIV to have significant healthcare-associated costs outside of the free-of-charge HIV care, primarily from costs related to transportation and accommodations [37]. However, our measure of per-visit costs does not support this finding. Our per-visit measure includes costs outside of the direct visit, like transportation and related accommodations. Still, no differences were found in the per-visit costs across the three HIV groups. In contrast, diabetic individuals reported higher per-visits costs for public clinic and hospital care. Shifting disease burdens towards chronic and historically underfunded diseases in LMICs, like diabetes, may be changing the landscape of health expenditure inequities.

The differences observed between virally suppressed and unsuppressed individuals in comparison to individuals without HIV indicate that the role of healthcare utilization on subsequent health outcomes is likely complex and dependent upon the type of services accessed. We observed that both of the HIV + aware groups reported more public clinic visits than the HIV-/status unaware group, but only the virally suppressed group displayed health gains across all four selected conditions. This may be because virally suppressed individuals have longer histories of regular interaction with the healthcare system compared to virally unsuppressed individuals. For instance, individuals who are virally unsuppressed may be earlier on in their treatment compared to those who have reached viral suppression. It is also possible that the healthcare interactions of virally unsuppressed individuals are on an acute, rather than preventive, basis. This is indicated by the higher number of hospital visits observed in virally unsuppressed individuals compared to HIV-negative individuals. In contrast, this was not observed in virally suppressed individuals. A lack of viral suppression could also indicate an inability to adhere to treatment. This inability to adhere to HIV treatment may translate into barriers in the management of other conditions. It is also possible that individuals who do not adhere to treatment may be less likely to utilize healthcare services as a whole.

Our results also highlight that individuals with diabetes are spending disproportionately more on healthcare than non-diseased individuals, a pattern not observed for individuals with HIV, a similarly healthcare-intensive condition. Despite high spending, diabetic individuals are not receiving the same health benefits as PLHIV. This could be attributed to a poorer overall baseline health status for diabetic individuals, as onset of T2D is associated with other poor health behaviors like sedentary lifestyles and poor eating habits [19, 38]. This is also indicated by the increased number of average hospital visits observed in diabetic individuals in our study. Spending on acute, emergency care may prevent affected households from spending on preventive care that would be more likely to influence long-term health outcomes. Supporting this idea, previous research in South Africa found a high incidence of catastrophic health expenditures among diabetic patients at public hospitals despite South Africa’s laws regarding free provision of care [36]. Combined, the results from diabetic and virally unsuppressed individuals in contrast to virally suppressed individuals underscore the potential importance of specifically primary and preventive care and warrants further investigation.

Our results differ from a previous study in South Africa that found high healthcare costs for PLHIV, both pre- and post-ART initiation [37]. Though PLHIV in our study were paying for healthcare, these costs did not differ from non-PLHIV on a per-visit basis indicating low-levels of expenditure-related inequities between the two groups. An additional study from South Africa specifically explored the differences in health expenditures between older adults living and not living with HIV. This study found no differences in median health expenditures or catastrophic health expenditures between the groups [39]. Our study aligns with this result and adds the important layer of healthcare utilization, allowing for a more contextualized look at healthcare expenditures. Our combined results suggest that that HIV-affected households may be spending more than unaffected households, likely attributed to a greater frequency of healthcare utilization rather than an inequity in care costs. This is supported by our results that show virally suppressed PLHIV had higher numbers of healthcare visits but similar per-visit spending compared to non-PLHIV.

Various studies have shown greater use of preventive healthcare services in PLHIV which further supports the idea that care-seeking PLHIV can overcome utilization barriers more easily than non-PLHIV. For example, studies from various African contexts have shown greater rates of uptake for contraceptives overall, long-acting contraceptives, births in formal facilities, and sleeping under bed nets for women living with HIV compared to women who are not [40–43]. These findings suggest that PLHIV seeking treatment may be exposed to more health education materials than those not living with HIV, thus helping to eliminate the barrier of health literacy. The converse hypothesis would be that PLHIV are more likely to experience any illness in comparison to those not living with HIV, thus, resulting in higher overall spend on healthcare. However, this pathway is unlikely if most observed PLHIV are adhering appropriately to prescribed ART regimens. Evidence from South Africa shows high rates of treatment adherence and viral suppression in adults on ART with optimal adherence rates ≥ 95% in 87% of monitored adults and viral suppression in as high as 94% of monitored adults [44–46]. However, there is evidence that adherence may be lower in older populations [47]. This converse hypothesis may be true for the group of diabetics. These individuals are spending more on healthcare, likely at hospitals, and likely due to increased illness. This may limit their overall exposure to preventive services and reduce their ability to pay for them within this low-resource setting.

A primary limitation of this work is its design as a cross-sectional and secondary data analysis. These results should be examined carefully as the direction of association is not known. As the HAALSI cohort was not constructed with this specific research question in mind, it is possible that our analyses were not powered appropriately and did not have sufficient sample size to answer our research questions. Additionally, it is possible that the health profile observed in this population of rural, older adults living with HIV is driven by a survival bias rather than the association between increased healthcare utilization and expenditure observed in our results. Because this population is made up of older adults, it could be that to survive to this age, the HIV+ adults needed to be physically fitter than their counterparts who did not survive. This could cause the population of people living with HIV to be made up of primarily those with a positive health profile whereas the HIV-negative population may include a greater diversity in levels of physical fitness and overall health. More detailed longitudinal research would be needed to explore the above limitations. The self-reported nature of the outcomes data used in our analysis also presents a limitation. Self-reported data, especially surrounding spending, is likely subject to recall bias and social desirability bias. Therefore, there may be over- or underreporting of healthcare spending. However, we would expect this bias to be non-differential across exposure groups and, thus, the relative differences are likely unaffected.

Our results are also impacted by the set of co-morbid health conditions available in the HAALSI dataset. These conditions, like obesity and dyslipidemia, often share common causes with diabetes which may explain the strong association observed here. Therefore, future research should incorporate an expanded set of health conditions to further explore this relationship. Finally, we were not able to account for the duration of infection of both HIV and diabetes due to a lack of data. Virally suppressed PLHIV may have longer overall infection durations and thus greater healthcare system exposure compared to unsuppressed PLHIV and individuals with diabetes. The latter individuals could have shorter infection periods, thus contributing to the observed healthcare outcomes. Overall, it is possible that duration of infection significantly impacts the presence of comorbidities, especially the duration of uncontrolled infection.

Despite the identified limitations, our study has multiple strengths. The first strength is the large sample size of older adults living with HIV included in our analysis. Using our expanded definition of HIV + status awareness, we were able to include data for 742 PLHIV, larger than many studies examining issues related to PLHIV. The availability of laboratory-testing for HIV status is another strength, as we were able to identify 205 individuals that self-reported as HIV-negative but tested positive for HIV and ART. This triangulation allowed us to reduce the effects of social desirability bias and stigma as they relate to willingness to disclose HIV status. We used multiple measures of healthcare utilization and expenditure to triangulate our findings and increase the robustness of our conclusions. The illustrative comparison with diabetes diagnoses also allowed us to further contextualize our findings.

## Conclusion

Our results suggest that older adults living with HIV may visit public clinics more often than their HIV-negative counterparts but may spend similarly on a per-visit basis. This provides preliminary evidence that the positive health outcomes observed among PLHIV in rural South Africa may be linked to differing patterns of healthcare engagement. The easy accessibility and low cost of HIV care in South Africa may facilitate these positive benefits. Overall, these results imply that increased healthcare spending associated with public clinic visits may be a possible pathway that benefits long-term health outcomes while increased healthcare spending associated with hospitalizations may not. Through our illustrative comparison between PLHIV and diabetics, we provide evidence that shifting disease burdens towards chronic and historically underfunded diseases, like diabetes, may be changing the landscape of health expenditure inequities. Older adults with diabetes spent disproportionately more on healthcare than their peers but still suffered worse health outcomes. Future studies are needed to confirm if increases in healthcare expenditure correlate to increased utilization of preventive services as well as to determine the types of healthcare services that low-income PLHIV and their households are spending on. This research could elucidate whether our results support a pathway where provision of free HIV treatment as an introduction to the health system promotes increased use of other critical, preventive health services.

### Electronic supplementary material

Below is the link to the electronic supplementary material.

Supplementary Material 1

## Data Availability

The datasets analyzed during this study are available in the Harvard Dataverse repository, 10.7910/DVN/F5YHML & 10.7910/DVN/CXXYUU [48, 49].

## List of Abbreviations

AHDSS: Agincourt Health and Demographic Surveillance System
ART: Antiretroviral therapy
HAALSI: Health and Aging in Africa: A Longitudinal Study of an INDEPTH Community in South Africa
HIV: Human immunodeficiency virus
PLHIV: People living with human immunodeficiency virus
SES: Socioeconomic status
