# Supplementary material for: Disease awareness and healthcare utilization in rural South Africa: a comparative analysis of HIV and diabetes in the HAALSI cohort
Source: BMC Public Health. 2023 Nov 8;23:2202. doi: 10.1186/s12889-023-17043-2 (PMC10634006; doi:10.1186/s12889-023-17043-2)
Supplement: Supplementary file 1 — Supplementary Material 1 [file 12889_2023_17043_MOESM1_ESM.docx]

**Supplementary Material**

*Figure S1.* Comparison plots of average annual household spending and household healthcare expenditure (as a percent of total annual household spending^b^) across five wealth indices


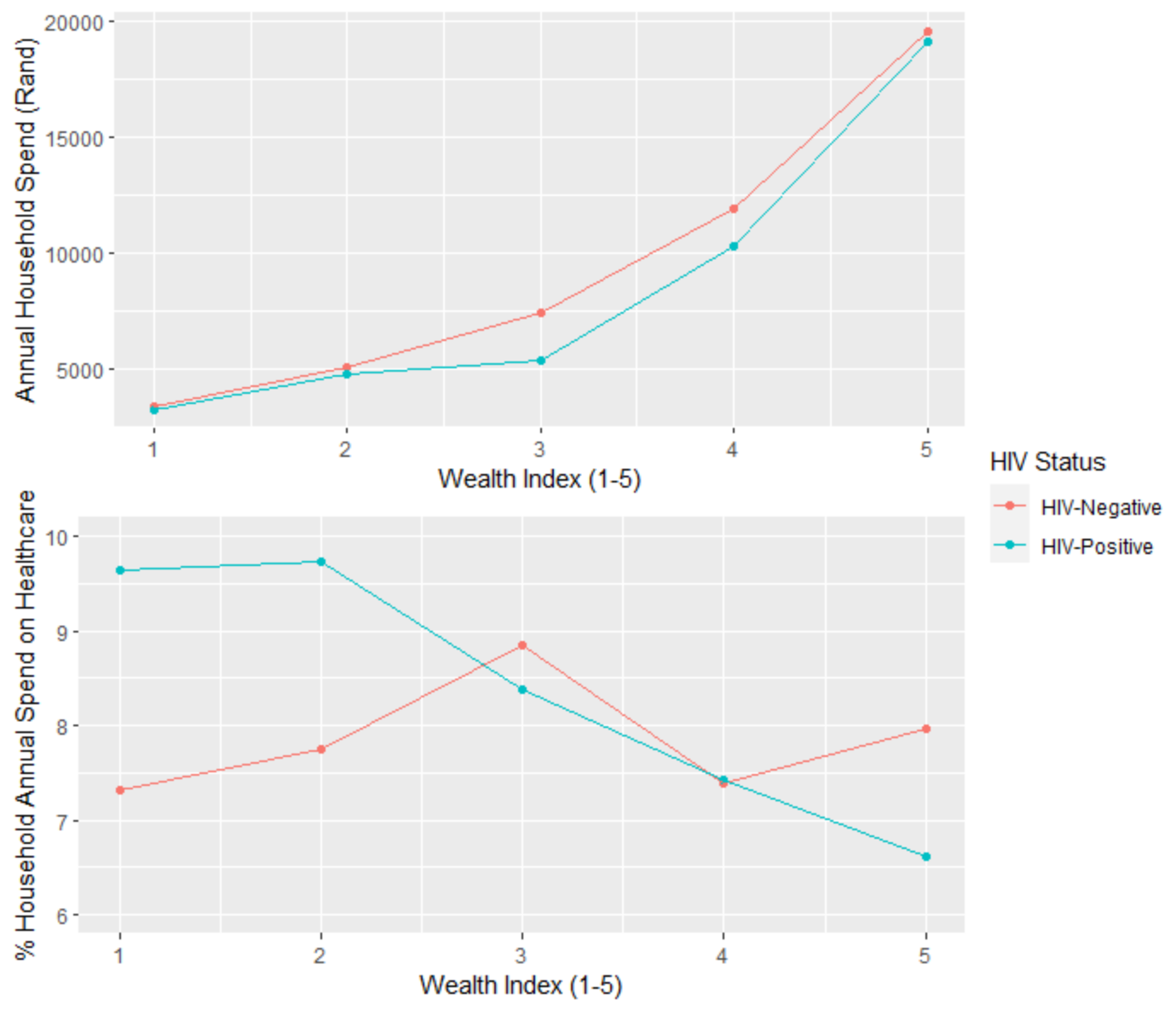


^a^Healthcare expenditure includes doctor’s fees, nurse’s fees, dentist fees, clinic fees, hospital fees, medications, bandages, supplies, and other pharmacy/chemist purchases ^b^Total annual household spending includes long distance travel, wedding expenses, birthdays, funerals, festivals, education, insurance premiums, home maintenance, vehicle service charges, taxes/fees/registration to government, healthcare, loan repayments, donations, and other expenses
